# Combining the potential of 3D printed buccal films and nanostructured lipid carriers for personalised cannabidiol delivery

**DOI:** 10.1007/s13346-023-01446-0

**Published:** 2023-10-30

**Authors:** Sadikalmahdi Abdella, Sangseo Kim, Franklin Afinjuomo, Yunmei Song, Richard Upton, Sanjay Garg

**Affiliations:** Centre for Pharmaceutical Innovation (CPI), Clinical and Health Sciences, University of South Australia, Adelaide, SA 5000 Australia

**Keywords:** CBD, Buccal film, 3D printing, NLCs, Design of experiments

## Abstract

Cannabidiol (CBD) has been recognized for its numerous therapeutic benefits, such as neuroprotection, anti-inflammatory effects, and cardioprotection. However, CBD has some limitations, including unpredictable pharmacokinetics and low oral bioavailability. To overcome the challenges associated with CBD delivery, we employed Design of Experiments (DoE), lipid carriers, and 3D printing techniques to optimize and develop buccal film loaded with CBD-NLCs. Three-factor Box-Behnken Design was carried out to optimise the NLCs and analyse the effect of independent factors on dependent factors. The emulsification-ultrasonication technique was used to prepare the NLCs. A pressure-assisted micro-syringe printing technique was used to produce the films. The produced films were studied for physicochemical, and mechanical properties, release profiles, and predicted in vivo performance. The observed particle size of the NLCs ranged from 12.17 to 84.91 nm whereas the PDI varied from 0.099 to 0.298. Lipid and sonication time positively affected the particle size whereas the surfactant concentration was inversely related. CBD was incorporated into the optimal formulation and the observed particle size, PDI, and zeta potential for the CBD-NLCs were 94.2 ± 0.47 nm, 0.11 ± 0.01 and − 11.8 ± 0.52 mV. Hydroxyethyl cellulose (HEC)-based gel containing the CBD-NLCs was prepared and used as a feed for 3D printing. The CBD-NLCs film demonstrated a slow and sustained in vitro release profile (84. 11 ± 7.02% in 6 h). The predicted AUC_0–10_ h, C_max_, and T_max_ were 201.5 µg·h/L, 0.74 µg/L, and 1.28 h for a film with 0.4 mg of CBD, respectively. The finding demonstrates that a buccal film of CBD-NLCs can be fabricated using 3D printing.

**Graphical Abstract:**

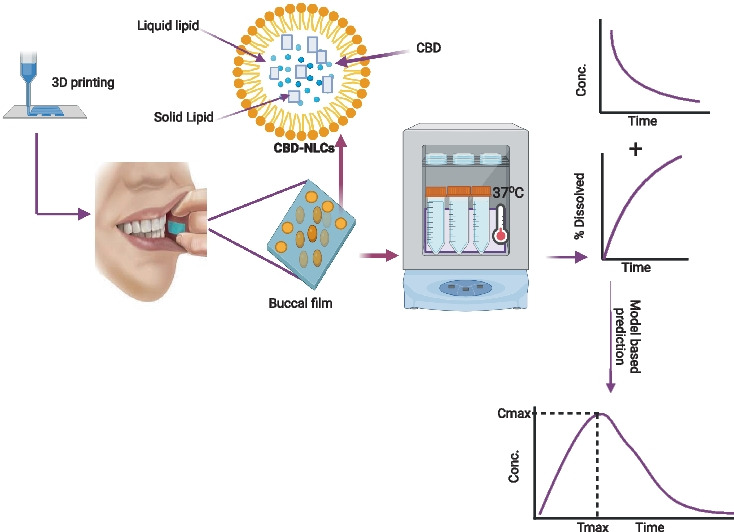

**Supplementary Information:**

The online version contains supplementary material available at 10.1007/s13346-023-01446-0.

## Introduction

Cannabidiol (CBD) is a non-psychoactive phytocannabinoid with several reported pharmacological effects including neuroprotection, cardioprotection, and anti-inflammatory effects [1, 2]. CBD has low toxicity, non-hallucinogenic effects, and is well tolerated at high doses, compared to other cannabinoids [3, 4]. Epidiolex^®^, the only marketed CBD monotherapy, has been approved by the European Medicines Agency (EMA) and FDA for tuberous sclerosis complex, Dravet syndrome, and Lennox-Gastaut syndrome associated seizures [5]. Additionally, a buccal spray called Sativex^®^ containing a 1:1 ratio of CBD and delta-9 tetrahydrocannabinol (THC) has been approved in over 25 countries for the treatment of muscle spasms related to multiple sclerosis [6].

Despite its potential advantages, CBD has unpredictable pharmacokinetics and low oral bioavailability (6%) mainly due to its significant presystemic metabolism, high lipophilicity (log P = 6.3), and low water solubility [7, 8]. Furthermore, CBD is unstable in gastric pH, highlighting the need to consider optional routes and drug delivery systems [9]. Several cannabinoids, including CBD, start to degrade at a temperature as high as 160 °C, resulting in decreased quantities [10].

Buccal drug delivery offers great advantages over other routes including oral and parenteral administrations [11]. It is a non-invasive, painless, and convenient method of drug administration [12]. Furthermore, this route bypasses both the enzymatic degradation in GI tract tract and hepatic first-pass metabolism, making it an ideal delivery route for drugs that undergo enzymatic degradation such as CBD [13]. It also allows direct systemic delivery of drugs due to the rich blood supply to the region. It is important to note that a buccal administration route is a viable option for patients who have difficulty swallowing, leading to improved treatment outcomes and better patient experiences. Buccal films are considered a patient-friendly dosage form due to their small size, ease of use, and storage. They can also be administered with minimal water, making them an ideal delivery system for many drugs [14]. Buccal films can also have multiple layers, allowing for sustained drug release within the oral cavity [15].

The utilization of nanoparticles based on lipids has been proposed as a compelling strategy to improve the solubility and bioavailability of drugs that have low water solubility, regulate release kinetics, and increase drug loading capabilities [16, 17]. They can be administered by a variety of routes including parenteral, mucosal, dermal, pulmonary, and topical [18–20]. NLCs are a newer type of lipid nanoparticle that contains a mixture of liquid and solid lipids, plus a surfactant at room temperature [21]. NLCs have many benefits over traditional carriers, including improved bioavailability and permeability, lower risk of side effects, and the ability to be produced on a large scale. In comparison to Solid Lipid Nanoparticles (SLNs), NLCs offer a greater drug-loading capacity for certain drugs and minimal drug expulsion during storage [22, 23].

3D printing, on the other hand, has gained significant attention as a progressive innovation in the pharmaceutical field and is expected to revolutionalize drug manufacturing [24]. Its use has expanded exponentially in recent years due to its potential advantages, including producing a personalized dose form with a specific shape, modified release kinetics, and color thereby ensuring patient-centricity [25–27]. Furthermore, 3D printing is able to produce a high-quality product, within minutes, saving time and resources [25].

3D printers produce dosage forms from digital models by gradually depositing material at precise locations in a layer-by-layer fashion [28–30]. The 3D printers commonly used in the pharmaceutical field are stereolithography (SLA) [31], inkjet, semi-solid extrusion, fused deposition modelling (FDM), binder-jetting, and selective laser sintering (SLS) printing [8, 9]. In semi-solid extrusion, objects are created by step-by-step deposition of layers of feed material, often paste or gel [10]. It offers several advantages, including the ability to print at low temperatures, fast printing speed, and meeting quality requirements [11].

The number of scientific articles on 3D printing for drug delivery has significantly increased over the last 10 years confirming the growing interest in the use of 3D printers for drug development [32]. Of note, the feasibility of 3D printing to produce tailored pharmaceutical dosage forms has also been proven by the FDA’s approval Spritam^®^ (levetiracetam) in 2015, 3D printed orodispersible tablet [33–35].

The advantages of 3D printing in developing personalised pharmaceutical formulations have been widely recognized [36, 37]. Customized dosage forms can be quickly produced by modifying their design using a computer-aided design (CAD) file. The customization considers individual patient needs including age, weight, organ function, disease condition, and patient preferences. Multiple drugs can also be printed in a single dosage form addressing the issue of polypharmacy and related medication adherence issues [38, 39].

Considering the challenges associated with oral CBD administration and the growing need to personalize therapy, we developed a buccal film of CBD using semi-solid extrusion 3D printing technology. Combining the advantages of buccal films, lipid-based nanoparticles, and 3D printing into a single system would improve the delivery of CBD. In addition, the NLC formulation was optimized using the Box-Behnken design. This design is a type of Response Surface Methodology (RSM) that is commonly used to optimise formulations as it requires fewer runs and less time compared to other methods [20]. RSM involves the application of mathematical and statistical techniques to analyse formulation obstacles and process parameters, facilitating the analysis and modeling of the relationship between the obtained response surfaces and the controllable input parameters [40, 41].

Buccal films containing SLNs of drugs were previously shown to improve the solubility and bioavailability of drugs [42, 43]. As far as our knowledge is concerned, this is the first study that reported NLCs-loaded buccal films. In this study, we developed a CBD buccal drug delivery system containing NLCs of CBD. The formulation could potentially improve the low bioavailability and variable pharmacokinetics of CBD. Polymers with mucoadhesive property were used to increase the bio-adhesiveness of the film. The film was characterized for physicochemical properties, mechanical properties as well as in vitro release properties. In vivo performance of the drug was predicted using a convolution method in R programming language.

## Methods and materials

### Materials

CBD was sourced from PM Separations in Queensland, Australia, and had a purity of ≥ 98%. Glyceryl distearate (Precirol^®^ ATO 5) was obtained from Gattefosse in Lyon, France. Hydroxyethyl cellulose NF was provided by Medisca (NY, USA). Sigma-Aldrich in New South Wales, Australia provided polyethylene glycol 400, Tween 80^®^, and liquid oil capric/caprylic triglycerides. Deionised water with a resistivity of 18.2 MΩ at 25 °C was used to prepare the formulations and all chemicals were of the highest commercial grade available.

### HPLC method for quantification of CBD

**Fig. 1:**
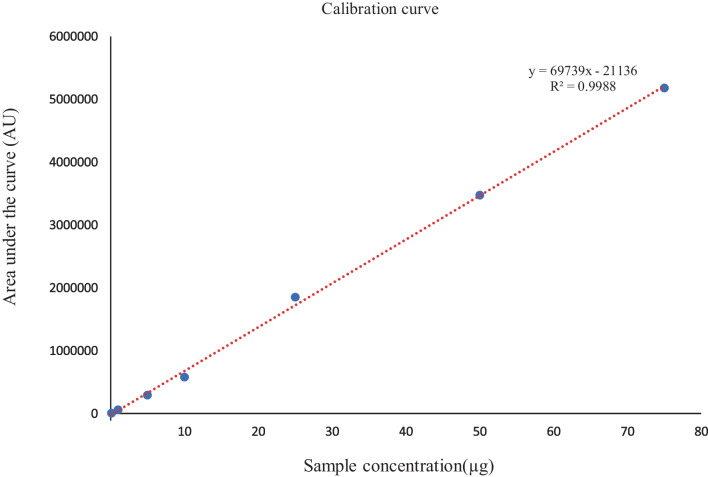
Calibration curve of CBD and regression equation

HPLC (Shimadzu Corporation, Kyoto, Japan) equipped with a degasser (DGU-20A3), an autosampler (SIL-20A HT), a pump (LC-20ADXR), and a photodiode array detector (PDA) (SPD-M20A) was utilized to analyse CBD. A Luna 5 µm C8(2) 100 Å column (250 × 4.6 mm) was used. The mobile phase consisted of acetonitrile and water (80:20 v/v). The flow rate and injection volume were 1.0 mL•min^−1^ and 10 μL. The peak was detected at 7.9 min with the help of a PDA detector using a wavelength of 210 nm. A calibration curve was constructed and used to quantify the amount of drug release over time (Fig. 1). The method was validated for determination of CBD. The performance parameters including linearity, accuracy, specificity, precision, and sensitivity (limit of detection and limit of quantitation) were determined according to International Conference on Harmonization ICH Q2 (R1) guidelines.

### NLCs preparation and optimization

Solid lipids including Gelucire 48/16, Precirol^®^ ATO 5, Stearic acid, Compritol^®^ ATO 888, Dynasan^®^ 116, and Dynasan^®^ 118 were considered for their suitability to prepare NLCs of CBD. Precirol^®^ ATO 5 was selected due to its relatively lower melting point (54 °C) and effectiveness to produce the best cannabinoid-loaded lipid nanoparticles [44]. Furthermore, Precirol^®^ ATO 5F has been shown to effectively mask the taste of bitter drugs [45]. Similarly, caprylic/capric oil was selected as a liquid oil due to better stability of CBD in medium-chain triglyceride. Calvi et al. demonstrated the absence of any lipid oxidation products when CBD was dissolved in medium-chain triglycerides (MCT) illustrating that MCT oil matrices were less prone to oxidative degradation compared to hemp seed oil or olive oil [46]. Tween 80^®^ was used as a surfactant due to its lower irritation to the cell membrane, low toxicity, widespread use in the pharmaceutical field and success in preparing NLCs [22, 47].

**Fig. 2:**
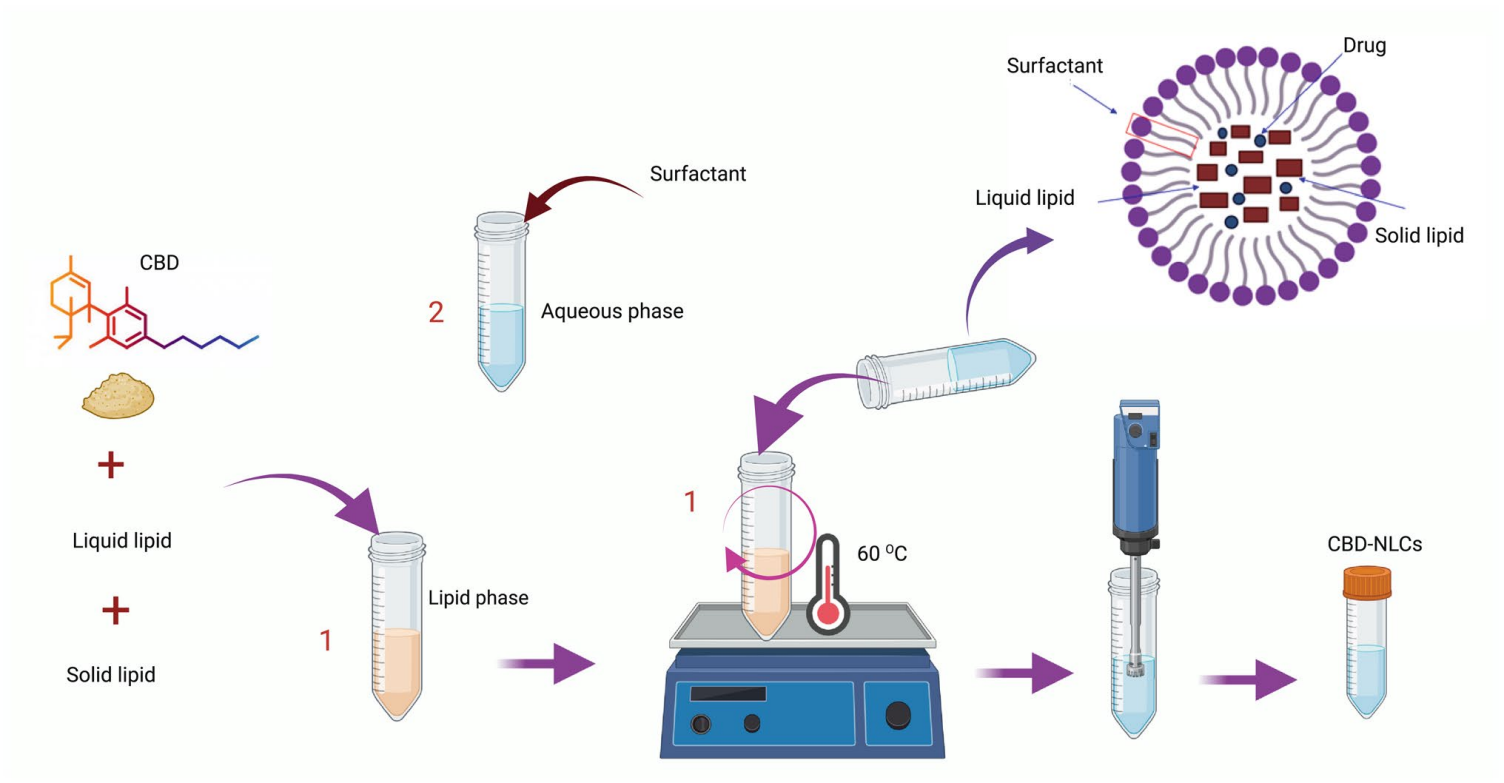
Preparation of CBD-NLCs. Created with biorender.com

The NLCs were prepared by hot emulsification-ultrasonication method [48]. Briefly, lipid phase (Precirol^®^ ATO 5 and Caprylic/Capric oil 70:30%w/w) was heated to 70˚C (5 °C above the melting point of Precirol^®^ ATO 5). The aqueous phase was simultaneously prepared by mixing the surfactant (Tween 80^®^) with de-ionised water and heating to the same temperature as the oily phase. Subsequently, the aqueous phase was poured into the lipid phase under continuous shaking and the mixture was exposed to ultrasonication (60% amplitude, 20 s on–off) (QSonica Q500, CT, USA) to form the NLCs (Fig. 2). The mixture was stored in a refrigerator (4 °C) prior to the preparation of the buccal film. To produce lyophilized NLCs, the blend was cooled in a freezer at − 80 °C for 1 h and then subjected to lyophilization using a freeze dryer (Lyph-Lock^®^ 6, Labconco, Kansas, USA) for 48 h at a pressure of 0.06 mbar and temperature of − 45 °C. This process was used to produce a blank dispersion (without CBD) and a dispersion of NLC containing CBD at a concentration of 2% (w/w). Table 1 provides details of the composition of these dispersions.

**Table 1:**
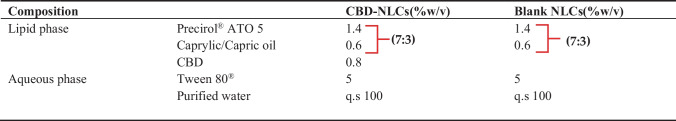
Placebo and CBD-loaded NLCs composition

Design of Experiments (DoE) was utilized to screen and optimize the concentration of different ingredients and processing parameters. The Three-factor Box-Behnken Design was selected for the optimisation of the formulation and analysis of the effect of independent factors on dependent factors, using the Design Expert software version 13. The Box-Behnken design was preferred due to its ability to analyse quadratic response surfaces and polynomial models with the minimum possible number of runs [49]. The studied independent variables were the total lipid concentration (% w/v TL), surfactant concentration (v/v %), and ultrasonication time (min) at three levels (− 1, 0, + 1). The dependent variables analysed were particle size (Y1) and polydispersity index (Y2) (Table 2). The ratio of solid to liquid lipid (oil) was kept constant at 70:30 throughout the study. Seventeen blank NLC formulations were prepared, and the optimised formulation was utilized to prepare CBD-loaded NLCs. The significance of the effects, lack of fit, and their interactions were evaluated using a significance level of 95% (α = 0.05) [42].

**Table 2:** Variables selected for the preparation of CBD-NLCs

**Factor**	**Level and code used**		
	Low (− 1)	Medium (0)	High (+ 1)
**Independent variables**			
X1 = Total lipid (% w/v)	1	3	5
X2 = Surfactant concentration (%v/v)	2.5	5	10
X3 = Ultrasonication time (min)	4	6	8
**Dependent variables**	**Constraints**		
Y1 = Particle size (nm)	Minimum		
Y2 = PDI	Minimum		

The generated quadratic model for the design expert generated 17 runs is shown below.

$$\begin{aligned}\text{Y}=\,&\text{F}_0+\text{F}_1\text{X}_1+\text{F}_2\text{X}_2+\text{F}_3\text{X}_3+\text{F}_{12}\text{X}_1\text{X}_2+\text{F}_{13}\text{X}_1\text{X}_3\\&+\text{F}_{23}\text{X}_2\text{X}_3+\text{F}_{11}{\text{X}_1}^2+\text{F}_{22}{\text{X}_2}^{2}+\text{F}_{33}{\text{X}_3}^{2}\end{aligned}$$

In the multiple regression equation, Y represents the dependent variable, d0 is the intercept, and d1 to d33 represent the regression coefficients calculated from the observed responses of the independent variables X1 to X3 at coded levels. X1 represents the solid-to-liquid lipid ratio, X2 represents surfactant concentration, and X3 represents ultrasonication time.

### In vitro characterisation of prepared CBD-loaded NLCs

#### Zeta potential, particle size, and polydispersity index

DLS was employed to determine the average polydispersity index (PDI), particle size, and zeta potential of the samples, using a zetasizer (Malvern Instruments, UK) at a temperature of 25 °C. A 100-fold dilution of all samples was prepared using deionized water and then injected into a disposable cuvette. The zeta potential was measured for both the optimized formulation and CBD-loaded NLC. All measurements were carried out in triplicate (*n* = 3) [50].

#### Entrapment efficiency (EE %) and drug loading (DL%)

The technique used for determining the entrapment efficiency (EE) and drug loading (DL) was based on ultrafiltration/centrifugation [31]. To achieve this, CBD-NLCs (0.5 ml) was introduced into Amicon^Ⓡ^ (50-KD cut-off) ultrafiltration devices and centrifuged at 3400 rpm for 30 min. The NLCs held on the filter were washed three times to eliminate any free drug, and the HPLC method described above (the “HPLC method for quantification of CBD” section) was used to determine the quantity of CBD in the filtered pool (free drug). Total amount of CBD was determined by first diluting the NLCs (50 µL) in simulated salivary fluid (X 20 dilution) and analysing using HPLC. Equations (1) and (2) were used to calculate the EE (%) and DL (%) respectively.

1 $$\mathrm{EE}\%=\frac{\text{drug amount(initial)}-\text{drug amount(free)}}{drug\;amount(total)}\times 100$$

2 $$\mathrm{DL}\%=\frac{\text{drug amount(total)}-\text{drug amount(free)}}{lipid\;amount(total)}\times 100$$

#### Desirability and optimization

The optimization of CBD-loaded NLCs involved the utilization of numerical optimization and the desirability function approach. The main aim was to obtain NLCs with the smallest possible particle size and PDI. To determine the optimal values for the independent variables, the desirability function method was employed. This approach entailed evaluating the desirability index for each response variable and then combining all response variables into a single desirability function that ranged from 0 to 1, indicating the ideal values of the independent parameters [51].

### Feed preparation and 3D printing of CBD-NLCs film

Polymers such as Polyvinylpyrrolidone (PVC), Hypromellose (HPMC_E50_), Poly(vinyl alcohol) (PVA) and Hydroxyethyl cellulose (HEC) alone and in combination were examined for 3D printing. HEC-based formulation resulted in a good film, upon visual inspection and was used for preparing CBD-NLCs loaded buccal film using 3D printing. Briefly, the gel was prepared by dissolving 8% of HEC (H) and 2.4% PEG (Mw ~ 400) in water. First, PEG was dissolved in water heated to 60 °C. The separately prepared CBD-NLC was added to the heated solution bit by bit under continuous stirring. Finally, HEC was added to the formulation and stirred until a uniform dispersion was formed (Fig. 4b).

**Fig. 3:**
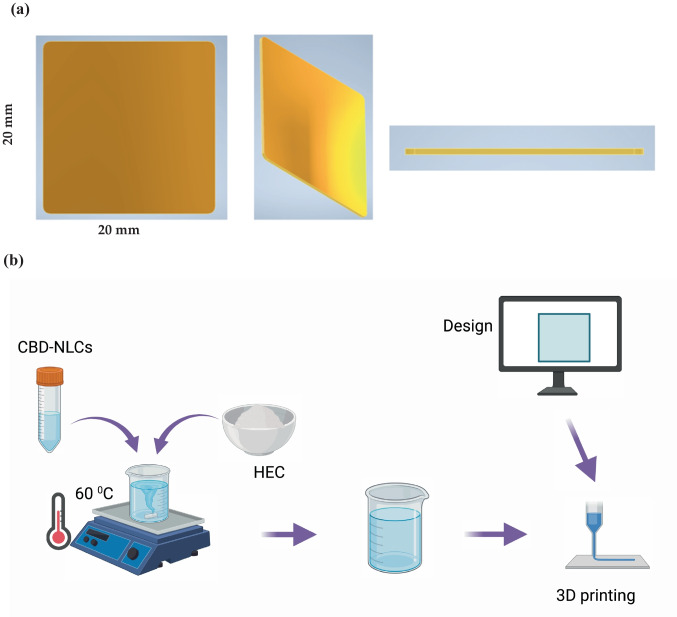
Design (**a**) and 3D printing of CBD film (**b**). Created using Biorender.com

A square film (20 × 20 mm^2^, thickness = 1 mm) was designed using Autodesk Inventor^®^ Professional 2021 software. The resulting designs were saved in stl format and converted into G-code files, which were readable by the 3D printer software. PAM (Bio X, Cellink, Gothenburg, Sweden) was used to manufacture the film. Approximately 2 mL of the formulation was loaded into the printer cartridge using a 5 mL syringe. Printing was carried out at a nozzle speed of 2 mm/s and a pressure of 90 kPa using a 25 G bioprinter nozzle. The films were subsequently dried for 48 h at room temperature, protected from light (Fig. 3a, b).

### Characterization of the optimised CBD-NLCs and 3D-printed CBD film

#### Physical appearance

Smoothness and homogeneity were assessed for the printed films, followed by the characterization of physicochemical properties and release kinetics. The thickness and weight of the films were determined after drying them at room temperature for 48 h using a digital micrometer and weighing balance, as outlined by Bala et al. [52].

#### Nanoparticle size recovery

The particle size recovery from the 3D printed film was evaluated in triplicate using zetasizer (Malvern Instruments, UK) at a temperature of 25 °C. Each 3D printed film (20*20 mm^2^) containing CBD was dispersed in 10 mL of deionized water, under constant stirring until its complete disintegration. Subsequently, the samples were filtered using 0.22 µm syringe filter and diluted 100-fold before injecting into a disposable cuvette for particle size analysis.

#### Mechanical characteristics and mucoadhesion determination

A texture analyzer (Stable Micro Systems, Godalming Surrey, UK) was used to evaluate the elongation at break and tensile strength (TS). The films were pulled apart, at the loading length of 200 mm, until breakage occurs by moving the upper clamp at a rate of 1 mm/s. The lower clamp remains stationary. The mucoadhesion was determined using a texture analyser as previously described by our group [27]. Briefly, the porcine mucosa was first mounted on the platform and the film was attached to a probe using a double-adhesive tape. The probe was then lowered at 0.5 mm/s and allowed to maintain contact with mucosa for 2 min. Lastly, the probe was withdrawn at 1 mm/s and the maximum force applied to completely detach the film from the buccal tissue (Fmax) was recorded. All the measurements were done in triplicate.

#### Fourier transform infrared spectroscopy (FTIR)

FTIR-attenuated total reflectance spectra of the CBD, Precirol^®^ ATO 5, lipid mix (Precirol^®^ ATO 5 + Caprylic oil), HEC, physical mixture (CBD, Precirol^®^ ATO 5, lipid mix and HEC), blank and CBD loaded film were obtained using FTIR spectrometer (Bruker, Massachusetts, USA). The spectra were recorded at room temperature in a range of 4000 to 450 cm^−1^ in transmittance mode using 4 scans per analysis at a resolution of 4.0 cm^−1^. A small portion of the films or powder was placed on ATR diamond crystal followed by application of force with the use of the clamp to ensure adequate contact of the sample with the crystal.

#### Differential scanning calorimetry (DSC)

DSC measurements of CBD, Precirol^®^ ATO 5, lipid mix (Precirol^®^ ATO 5 + Caprylic oil), HEC, physical mixture (CBD, Precirol^®^ ATO 5, lipid mix and HEC), blank and CBD loaded film were taken in Discovery DSC 2920 (TA Instruments (New Castle, USA) calibrated with an indium standard. Samples weighing 4.0 ± 0.5 mg were put in aluminum pans followed by recording of thermal profiles by heating the samples from 25 to 250 °C at a rate of 10 °C/min while continuously flowing nitrogen gas.

#### Scanning electron microscopy (SEM)

The morphology of the films and pure drug was evaluated using a Zeiss Merlin Field-Emission Dispersive X-Ray Spectroscopy (Jena, Germany) operating at an accelerating voltage range of 2–5 kV, after sputter-coating with platinum.

#### Film thickness and dry weight

Thickness of the film was determined by measuring five locations (four corners and one center) using a digital micrometer (ID-S1012, Mitutoyo, Japan) as described by Bala et al. [53]. Dry weight of the film was determined by randomly cutting four pieces (0.64 cm^2^) and weighing them using a digital balance.

#### Surface pH

The surface pH of each film (*n* = 3) was measured by adding a drop of MilliQ water to the surface and measuring with a pH meter (Orion Star A121, Thermo Scientific, USA) [52].

#### Folding endurance

The folding endurance was assessed by continually folding each film (*n* = 3) at the same spot until breakage and recording the total number of folds.

#### Drug loading

To determine the drug loading, films (20*20 mm^2^) (*n* = 3) were placed in a Falcon tube containing a hydro-alcoholic solution (10 mL, 50:50 v/v) maintained at 37 °C for 1 h. The solution was then centrifuged at 3000 rpm for 5 min, filtered, and analysed using HPLC.

#### In vitro release experiments

The method used to determine the in vitro release of CBD from the buccal film was similar to the one reported by our research group earlier [54]. The films (*n* = 3) were placed in a Falcon tube with 10 mL of simulated salivary fluid (SSF) and kept in a shaking water bath (Julabo SW22, Germany) at 37 ± 0.5 °C while being stirred at 50 rpm. At fixed time intervals of 10, 20, 30, 45, 60, 90, 120, 180, 240 and 360 min, 1 mL aliquots of the sample were withdrawn and an equal volume of fresh SSF was replaced. HPLC was used to analyse the drug content in the withdrawn samples after filtering the samples with 0.45 µM syringe filters.

#### Mathematical modeling of drug release profiles and prediction of in vivo performance

Several mathematical models were fitted to the drug release data obtained from in vitro release studies in the simulated salivary medium using a DD solver add-in in Microsoft excel [55] (Supplementary Table S1). The adjusted R2, the Root Mean Square Error (RMSE), and the Akaike Information Criterion (AIC) were used to evaluate the goodness of fit.

A convolution approach was used to predict the in vivo performance of the film, as described by our previous report. A convolve function in R programming language was used to perform the convolution [54].

## Results and discussion

This study reports the 3D printing of CBD-NLCs-loaded buccal film using the PAM 3D printing technique. The NLCs were prepared by mixing solid lipid, surfactant, and liquid lipid using a hot emulsification-ultrasonication technique. The choice of lipids and surfactant was based on previous reports and the optimal formulation of NLCs was determined using the Box-Behnken design. CBD was incorporated into the optimized NLCs. The CBD-loaded NLCs were mixed with polymeric formulation (8% HEC and 2.4% PEG) to prepare a gel used as a feed for the 3D printing of the films.

### Experimental design and characterization of NLCs

Box-Behnken design with triplicates at the central point was carried out to analyse the influence of different factors: X1 total lipid (%), X2: surfactant concentration (%), and X3: sonication time(min) on NLC formulations.

#### Impact of independent factors on particle size (PS)

The size of nanoparticles has been shown to influence the optimal interaction with buccal mucosa [56]. The observed particle size ranges from 12.17 nm (SA12) to 84.91 nm (SA11) as shown in Table 3.

**Table 3:**
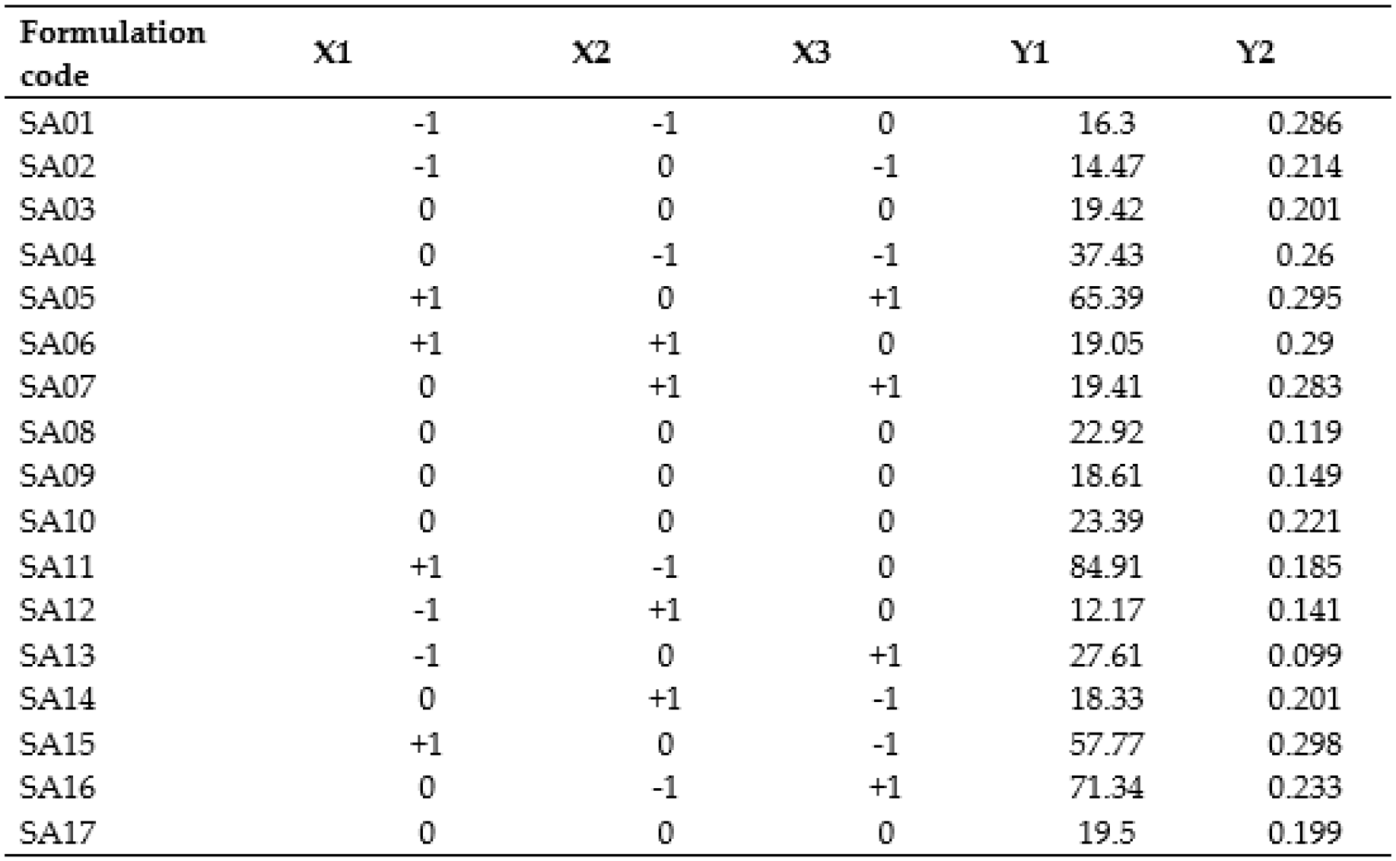
The composition and the measured responses of NLCs

X1 = Total lipid (% w/v), X2 = Surfactant concentration (%v/v), X3 = Ultrasonication time (min), Y1 = Particle size, Y2 = Polydispersity index

**Fig. 4:**
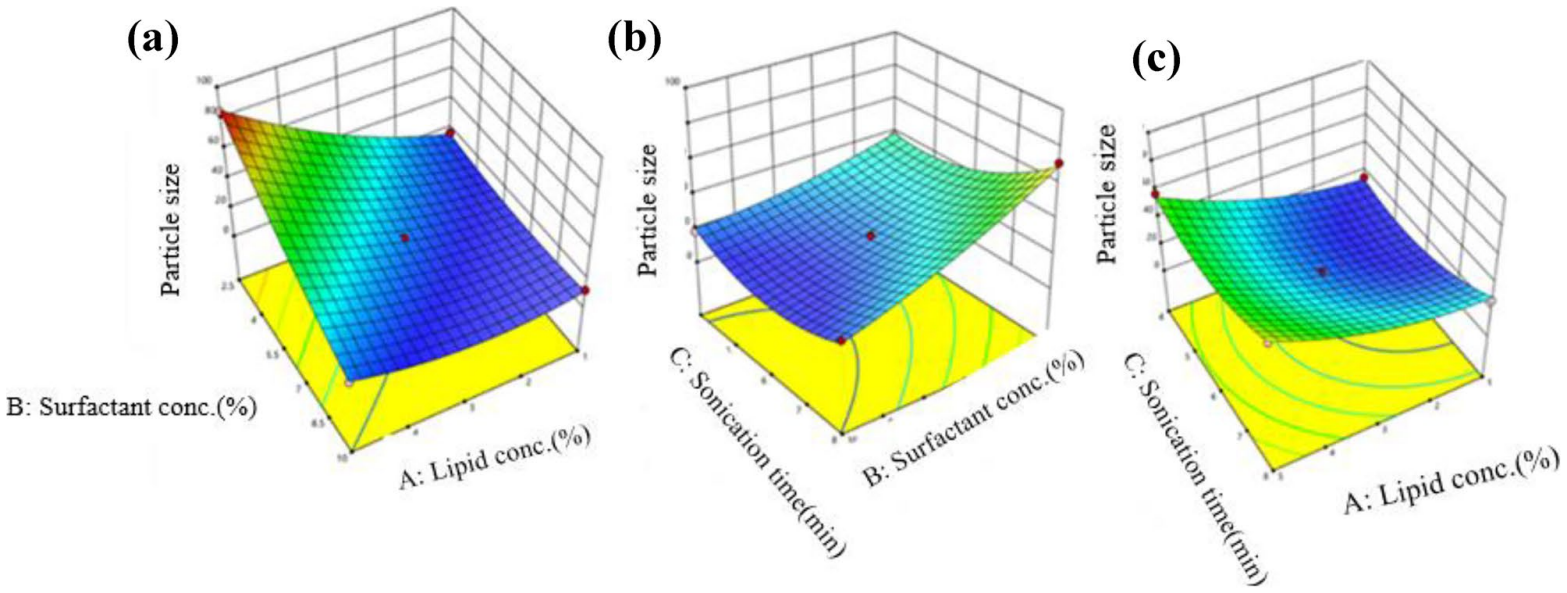
3D response surface plots illustrating the impact of independent factors on particles size

The ANOVA test was conducted to assess the impact of independent variables on the particle size of CBD-NLCs, and the quadratic model demonstrated a high level of significance with a narrow gap between predicted *R*^2^ (0.9396) and adjusted *R*^2^ (0.9865), and adequate precision (36.6857) (Table 4). The lack of fit was not significant (*p* > 0.05). Except for the interaction of lipid concentration and sonication (*p* > 0.05), all three independent variables and their interactions had a significant effect on the particle size of the NLC formulation (Fig. 4). Factor X_1_ (solid lipid amount), X_3_ (sonication time), X_12_, X_22_, and X_32_ had a positive effect on PS, whereas X_2_ (surfactant concentration) had a negative effect. The final equation, which is a combination of coded factors, confirms the result.

**Table 4:** Regression analysis of the dependent variables using the best fitting model

**Response**	**Model**	***R***^**2**^	**Adjusted *R***^**2**^	**Predicted *R***^**2**^	**Adequate precision**	**Significant terms**
**Y1: PS**	Quadratic	0.9941	0.9865	0.9396	36.6857	X_1_,X_2_,X_3_,X_1_X_2_,X_2_X_3_,X_12_,X_2_^2^,X^2^
**Y2: PDI**	Quadratic	0.5535	0.2856	− 0.4008	5.9882	

*Y*_*1*_ Particle size (nm), *Y*_*2*_ Polydispersity index

$$\begin{aligned}\text{PS}=\,&20.77+19.57\text{X}_1-17.63\text{X}_2+6.97\text{X}_3-15.43\text{X}_1\text{X}_3\\&-1.38\text{X}_1\text{X}_3-8.21\text{X}_2\text{X}_3+3.83{\text{X}_2}^2+8.51{\text{X}_1}^{2}+12.03\end{aligned}$$

The positive effect of the total lipid on the size of the nanoparticles could be due to the increase in the viscosity of the formulation that in turn reduces the effectiveness of particle-breaking (sonication) processes [57]. The increased particle size with the increase in the amount of total lipid could also be attributed to other reasons such as aggregation between lipid particles, increased chances of collision as well as inadequate surfactant amount to cover the lipid particle’s surface [58]. The finding agrees with the work of Kim et al. where NLCs of imiquimod with higher lipid concentration resulted in larger particle size. A similar effect of lipid amount on particle size was illustrated by Jain and colleagues [59] showing that the mean particle size of the prepared NLCs was significantly affected by the amount of total lipid. A counter effect of surfactant concentration on particle size was observed, where increasing the surfactant concentration produced a smaller particle size. High surfactant concentration was illustrated to decrease surface tension, thereby stabilising the surface during homogenisation and preventing particle agglomeration which in turn leads to the production of smaller particle size [60, 61]. Kim et al. [58] and Taha et al. [62] also reported similar findings. Sonication time showed biphasic responses indicating optimal sonication time was required to produce particles of the desired size as illustrated in Fig. 3b and c. The size of particles size decreases as the ultrasonic power increases however, excessive ultrasonic power promotes excessive growth of particles [63, 64].

#### Impact of independent factors on PDI

**Fig. 5:**
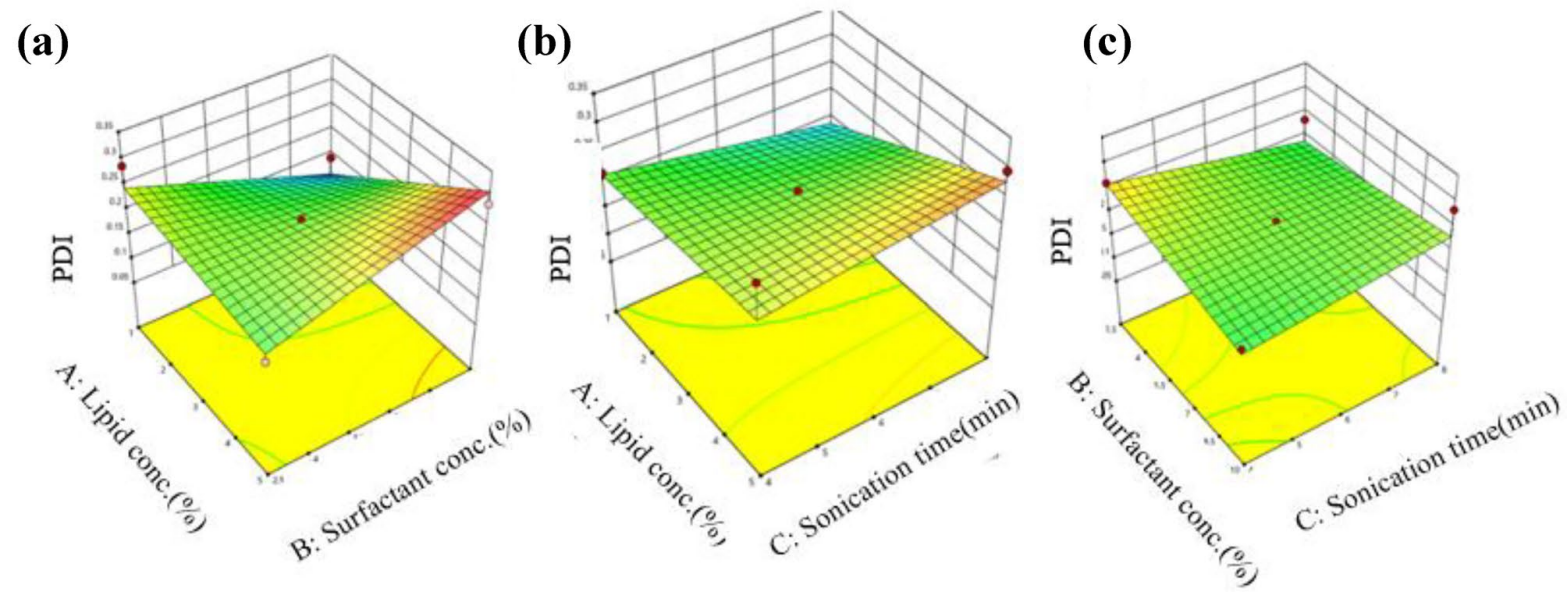
3D response surface plots illustrating the impact of independent factors on polydispersity index (PDI)

The PDI of the prepared NLCs varied from 0.099(SA 13) to 0.298(SA 15) as shown in Table 3. PDI gives information about the uniformity of the prepared nanoparticles. PDI values range from 0 to 1, 0 representing a perfectly homogenous system whereas 1 indicates a highly polydisperse system [65]. The low PDI values (< 0.3) confirm the uniform distribution of particle size in the NLCs formulation. Values closer to 0 assure the homogeneity of the formulations. The ANOVA test using a quadratic model revealed that the model was insignificant (*p* = 0.1484) with adequate precision of 5.9882. From the equation, there is a trend towards increased PDI with increasing total lipid amount and decreased PDI with increasing surfactant concentration and sonication time (Fig. 5).

$$\begin{aligned}\text{Y2}=\,&0.2161+0.0410\text{X}1-0.0061\text{X}2-0.0079\text{X}3\\&+0.0625\text{X}1\text{X}2+0.0280\text{X}2\text{X}3+0.0272\text{X}1\text{X}3\end{aligned}$$

**Fig. 6:**
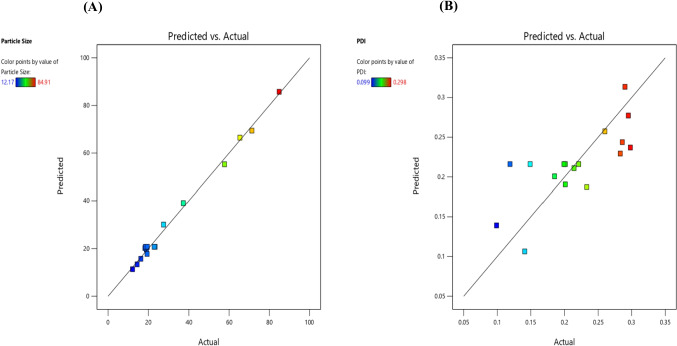
Correlation plot between actual and predicted **a** particle size (PS) and **b **polydispersity index (PDI)

**Fig. 7:**
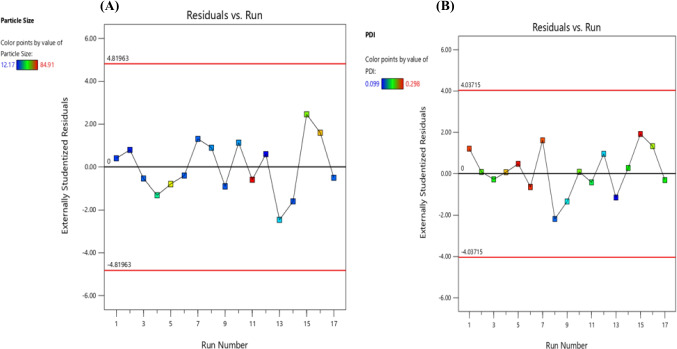
Residual plot between the run number and the residuals for **a** particle size (PS) and **b **Polydispersity index (PDI)

A quantitative comparison between predicted and actual values for Y1 and Y2 is illustrated by linear correlation plots with *R*^2^ of 0.9941 and 0.5535 respectively (Figs. 6A and 5B). Moreover, the reliability of dependent variables was tested using a residual plot between the run number and the residuals in Fig. 6a and b, respectively. All the data points lay within a 95% confidence interval as illustrated by the vertical spread of the studentized residuals from bottom-to-top, implying that (Fig. 7A, B).

### Validation of the model and selection of the optimised NLCs

Adequate precision, an estimate of signal-to-noise ratio, was used to adopt the most fitted model. Adequate precision values greater than 4 suggest that the model can explore the experimental design space. Also, the maximum *R*^2^ value was considered to choose the model. Minimum PS(Y1) and PDI(Y2) were considered to select the optimised formula. The software suggested optimised NLCs with a desirability value of 1.000. A formula comprising 2% Total lipid (X1), 5% surfactant concentration(X2) and 4.5 min sonication time(X3) was suggested as the optimal formulation by the desirability function. The measured variables were 16.5 (± 0.13) nm and 0.221 (± 0.006) for PS and PDI respectively. The % error was small as shown in Table 5.

**Table 5:** Predicted and observed responses of optimized NLCs

**Variables**	**Values**	**Responses**	**Predicted value**	**Actual value**	**Error%**
X1	2%	Y1: Particle Size (nm)	15.8	16.5	4.4%
X2	5%	Y2: Polydispersity index	0.231	0.221	4.3%
X3	4.5 min				

**Fig. 8:**
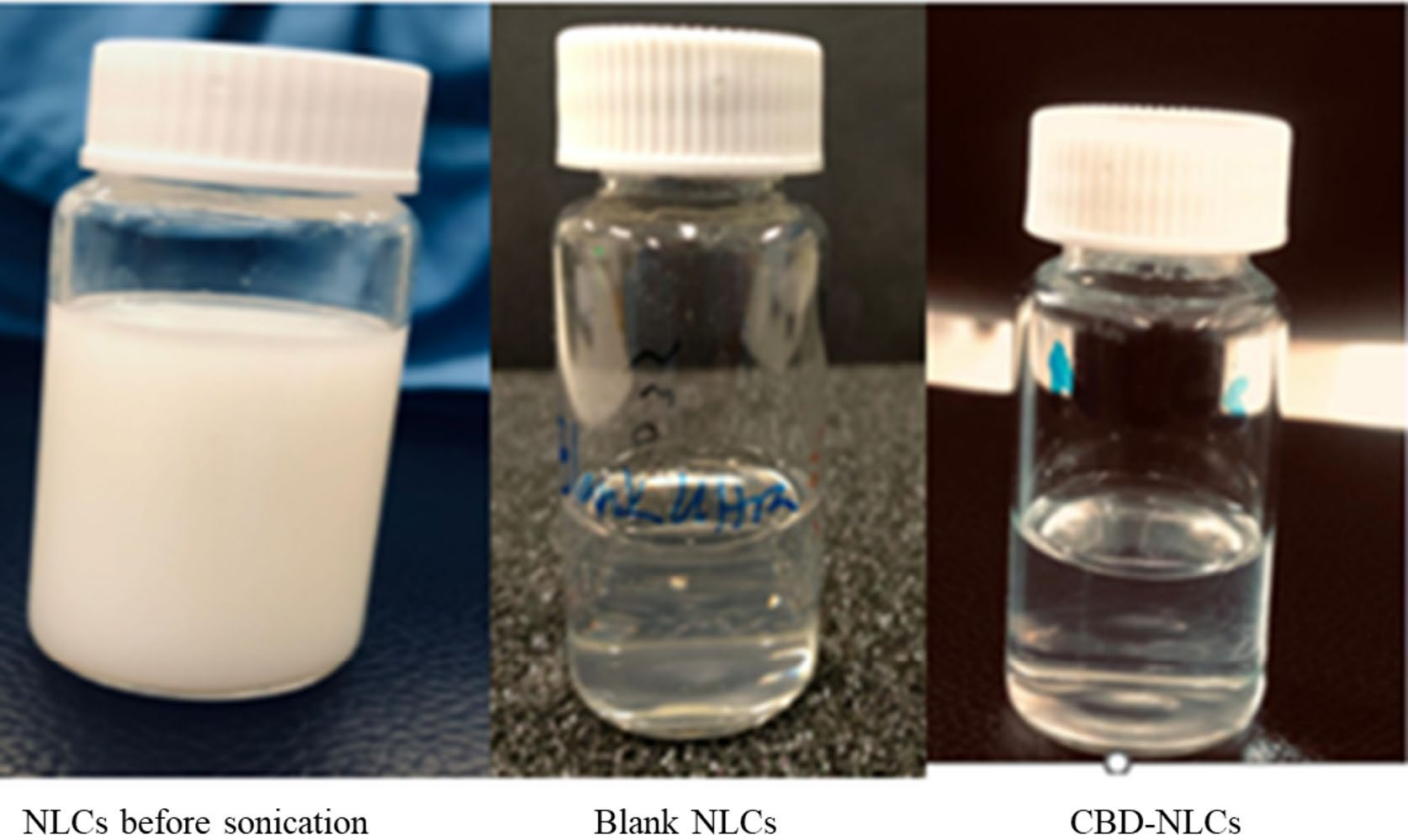
NLCs formulation and CBD-NLCs

The optimised NLCs were used to load CBD (Fig. 8). With CBD inclusion, the particle size increased to 94.2 ± 0.47 nm which is in the range of particle size recommended for drug delivery to biological cells. Particles less than 10 nm was illustrated to be cleared by the kidney whereas particles greater than 200 nm can be easily recognized by the mononuclear phagocyte system [66, 67]. The PDI and zeta potential of the CBD-NLCs was 0.11 ± 0.01 and − 11.8 ± 0.52 mV. The drug loading and entrapment efficiency were 0.83 ± 0.008% and 82.82 ± 0.77% respectively. The result was consistent with previous studies that reported higher entrapment of CBD in NLCs [31]. Several previous studies [68, 69] reported the inclusion or increasing the concentration of the drug increases the particle size of the nanosystem. This result could be related to an increase in the viscosity of the system following the addition of the solid phase in the lipid phase [70].

### Characterisation of the 3D printed CBD-NLCs film

#### Physical appearance

**Fig. 9:**
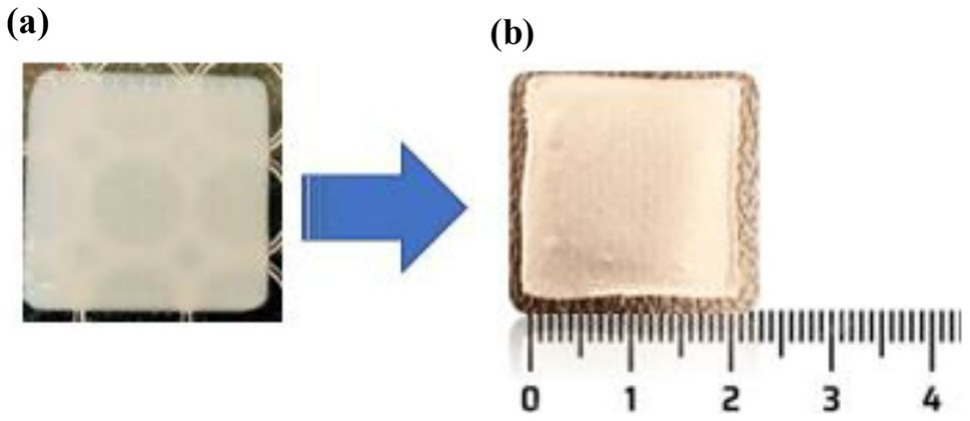
3D printed film (20*20 mm^2^) **a **wet film and **b **dry film

From the screening of mucoadhesive polymers including PVA, HPMC, and HEC either alone or in combination, HEC-based gel resulted in smooth and flexible printed films upon physical examination and visual inspection (Fig. 9). HEC-based gel was also reported to exhibit excellent printability and mucoadhesion [71]. A plain film with a 100% infill pattern was printed showed no sign of drug crystallisation.

The thickness of the film was 0.284 ± 0.009 mm, which lies in the ideal thickness range for buccal films [72]. Suitable thickness aids comfortable application of the film and determines the quantity of drugs [73]. The average weight of the film (0.64 cm^2^) was 0.14 ± 0.008 g. The weight variability was low as illustrated by low standard variation.

#### Particle size recovery

Recovery of particle size of nanocarriers after disintegration of the 3D printed film in water was determined using zetasizer. The particle size of the redispersed system was 183.7(PDI = 0.3). The result revealed an increment in particle size of the redispersed system compared to the CBD-NLCs before being dispersed in the polymer solution. This could be due to protective layer formed around the lipid nanoparticle by polymers. Freitas and colleagues illustrated that carbohydrates can form a thick protective layer around the lipid nanoparticles which protects them against the mechanical stress and heat stress during spray drying. They showed that higher concentration of carbohydrate such as mannitol resulted in an increased particle size upon redispersion in water [74].

#### Texture analyser

**Fig. 10:**
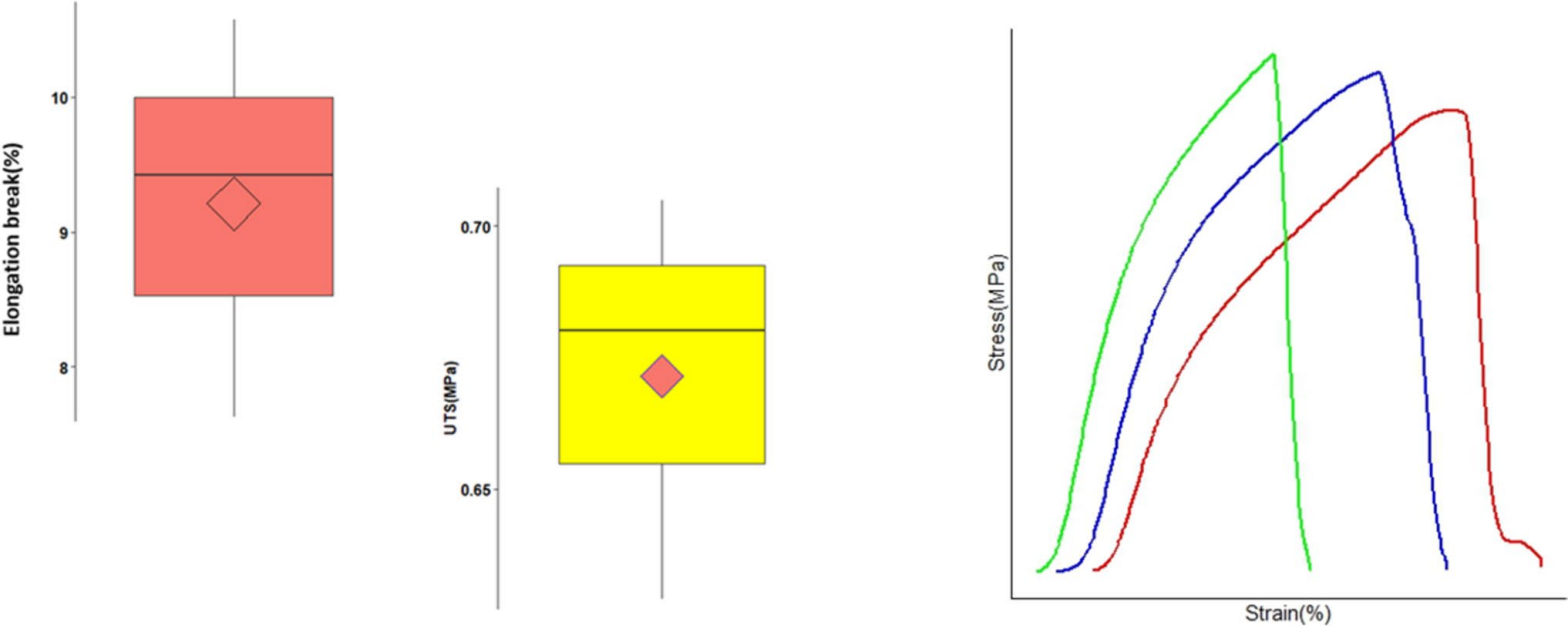
Tensile strength and Elongation break (%) of CBD-NLCs 3D printed films as box plots (*n* = 3)

The mechanical properties of the films were evaluated to ensure handling without breaking. Tensile strength refers to the traction that can be applied before the film breaks while elongation helps to assess the brittleness of the films [43]. Percent elongation and tensile strength of the drug-loaded 3D printed films were determined using a texture analyzer. The tensile strength and percent elongation were 0.67 ± 0.04 MPa and 9.2 ± 1.5% (Fig. 10). The tensile strength and elongation break of blank film was 1.26 ± 0.43 MPa and 4.4 ± 0.7% respectively. The introduction of CBD, which is lipophilic in nature, might have contributed to the decrease strength and improved elongation.

The result of mucoadhesion test using porcine buccal mucosa revealed that both blank and CBD loaded films have mucoadhesion capacity. The calculated DFmax for blank film and CBD loaded was 0.16 ± 0.03 N and 0.14 ± 0.02 N respectively. The slightly lower mucoadhesion of the CBD loaded film could be related to the oily nature of CBD. Previous studies have shown that HEC has strong mucoadhesive property [75].

#### Surface pH

The pH of the printed film was 5.9 ± 0.06. Oral films should have a neutral pH or close to a pH value of 7. This is important to avoid irritation to the oral mucosa. Of note, it might also affect the dispersion, taste as well as the release of the drugs [52, 73].

#### Drug loading

The drug content for the printed film (20*20 mm^2^) was determined after dissolving the films in a 10 mL of hydro-alcoholic solution (50:50 v/v) maintained at 37 ^O^C for 1 h. The sample was centrifuged as described above ("Drug loading") and supernatant was collected and analysed by HPLC ("NLCs preparation and optimization" section). The achieved drug content was 0.4 ± 0.03 mg for the 20*20 mm^2^. The dose can easily be tailored to cater for individual patient’s requirements by changing thickness and size of the film.

#### ATR-FTIR spectroscopy analysis

**Fig. 11:**
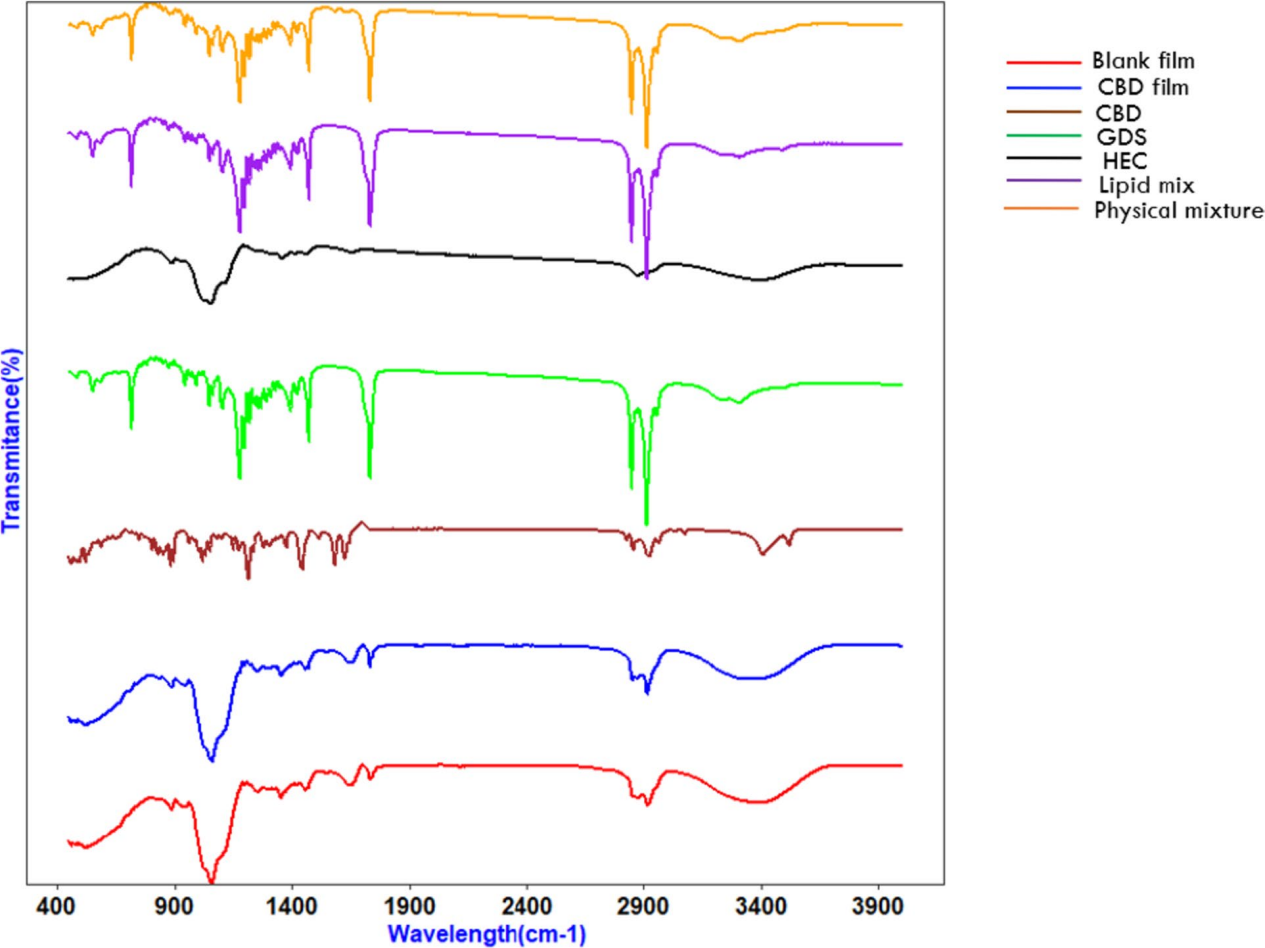
FTIR spectra of blank film, drug-loaded film, and their components

The study assessed potential interactions between CBD and the components of a film using infrared analysis (Fig. 11). The CBD spectrum displayed distinct bands, with the highest at 3519.30 cm^−1^ and 3406.56 cm^−1^ due to O–H stretching, bands in the range of 3100–2600 cm^−1^ caused by symmetric and asymmetric C–H stretching, two bands at 1622 and 1581 related to C = C stretching, and bands at 1442 cm^−1^ (C–H bending) and 1213 cm^−1^ (C–O stretching) [76]. In HEC, a characteristic peak for the stretching vibrations of saturated C–H was observed at 3371 cm^−1^ and 2888 cm^−1^, while the band at 1061 cm^−1^ was due to the stretching vibration of ether (C–O) [77]. The results of the FTIR analysis on the physical mixture of the film components showed peaks corresponding to Precirol^®^ ATO 5. The peaks of CBD were possibly concealed by the peaks of the polymer and Precirol^®^ ATO 5. The FT-IR spectra of the blank and drug-loaded film were similar, indicating that CBD was successfully incorporated into the film polymers without any interaction. Of note, this technique is not robust enough to prove encapsulation due to overlap of peaks.

#### DSC studies

**Fig. 12:**
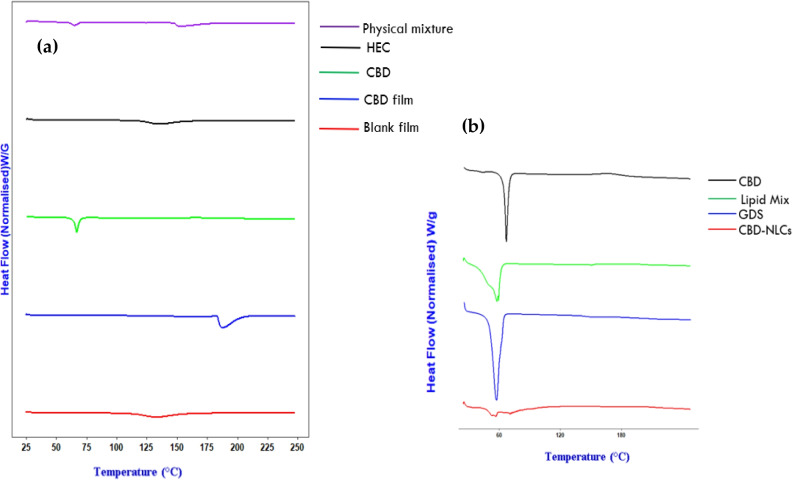
DSC thermograms of CBD film and CBD-NLCs and their components

**Fig. 13:**
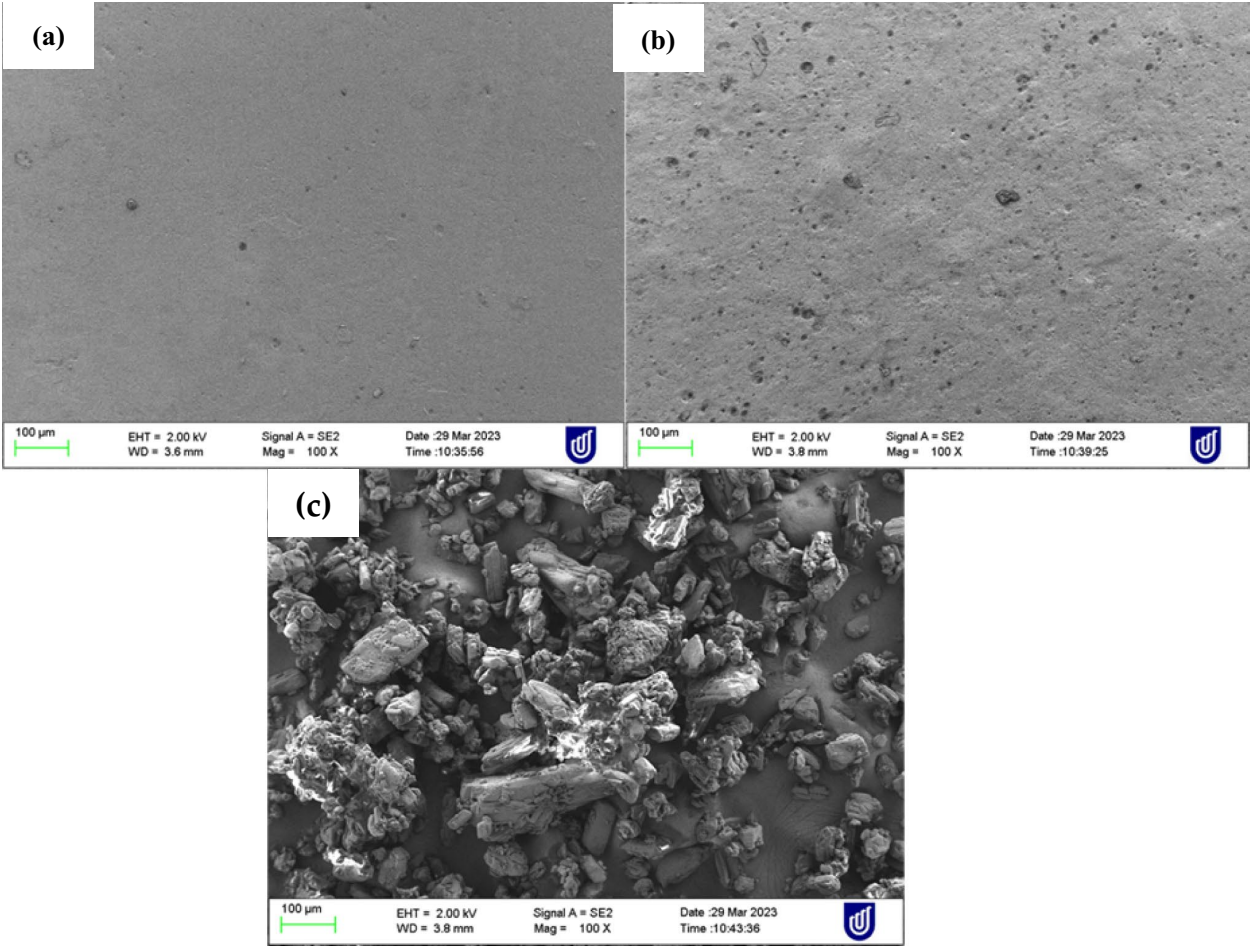
SEM of surface of **a **drug-loaded film **b **blank film and **c **pure drug

The crystallinity of CBD could affect both drug encapsulation and its release from the film. The DSC was evaluated for the film and NLCs components separately as illustrated in Fig. 12. A melting point peak was observed at 66.9 °C for pure CBD as reported in the literature [78] (Fig. 12a, b). No melting peak of CBD was observed for both CBD-NLCs and CBD film showing that either CBD has dissolved or in amorphous state. Similar finding was reported by Morakul and collegues [79]. Furthermore, the endothermic peak of Precirol^®^ ATO 5 was observed at 57.2 °C in lipid mix and pure Precirol^®^ ATO 5 [31, 80]. The peak of the lipid mix was less sharp than the solid lipid (Precirol^®^ ATO 5) which could be due to reduced crystallinity when melted with liquid oil (Fig. 13b). The glass transition temperature for pure HEC was observed around 143 °C (Fig. 13a) consistent with previous reports [81, 82]. No peak of CBD was observed in drug-loaded film as well as CBD-NLCs confirming the drug was not in crystalline state anymore.

#### Scanning electron microscopy (SEM)

Results from the SEM showed that pure CBD appeared as an irregular shaped crystals similar to a previous report [83]. The surface morphology of both blank films (Fig. 13a) and drug-loaded (Fig. 13b) was smooth indicating the drug was evenly distributed through the system. Nevertheless, the surface of CBD film was smoother compared to the blank which might be due to the surface being packed with tiny particles of the CBD.

#### In vitro drug release study

**Fig. 14:**
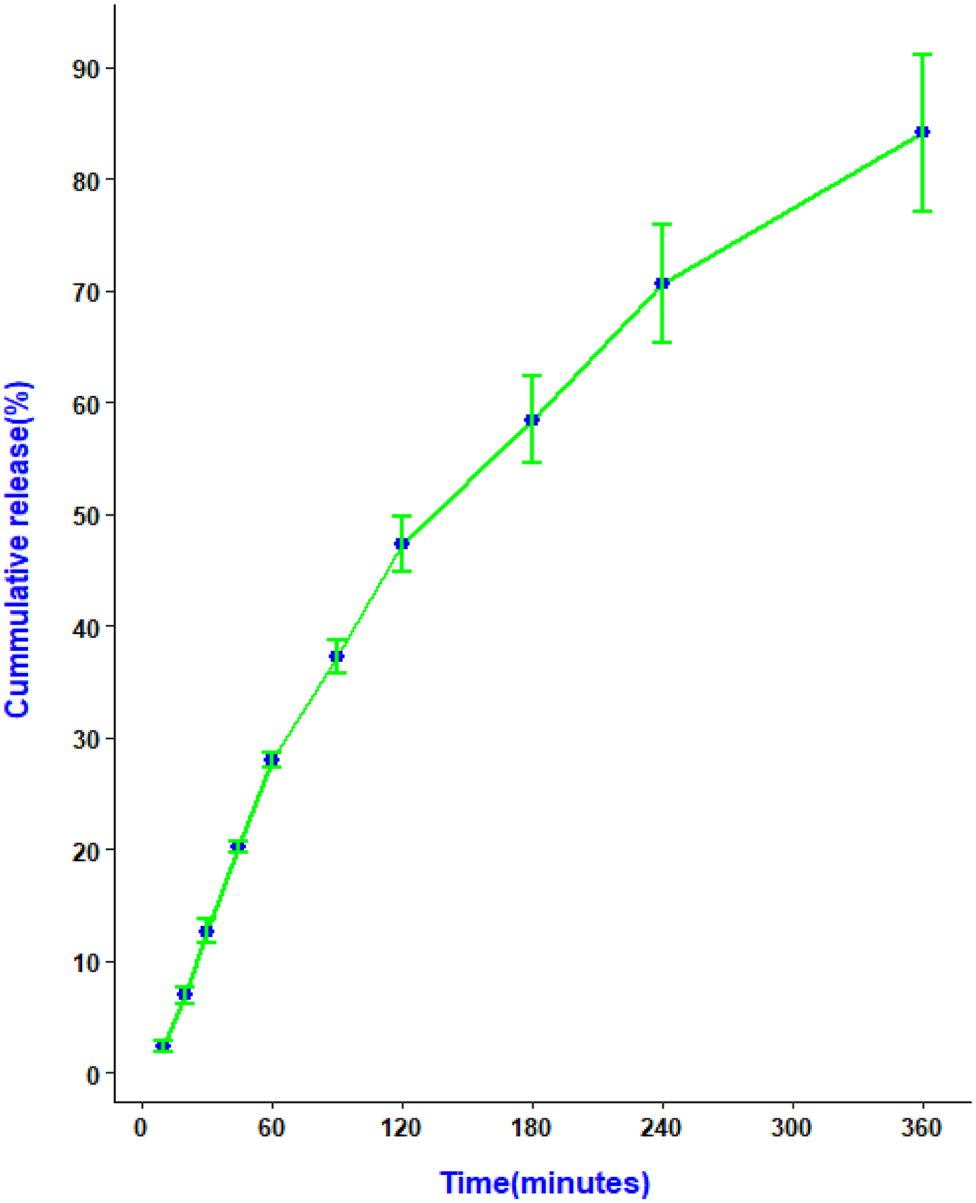
Cannabidiol (CBD) release profiles from CBD-NLC film (*n* = 3)

The release of the drug from the films was evaluated in a falcon tube filled with 10 mL artificial saliva adjusted to 37 °C for 6 h, as illustrated by our previous work [54]. The result of the in vitro release of CBD film is shown in Fig. 14. The release profile showed a slow and sustained release of CBD from the film (84. 11 ± 7.02% in 6 h). Similar release pattern was reported for Dexibuprofen-Loaded Nanostructured Lipid Carriers [84]. The release of drug particles on the surface of the NLCs during the first hours might have contributed to the relatively faster drug release in the initial phase [85, 86]. Several factors including production temperature, type and concentration of the emulsifier, the production techniques, and partition coefficient of drugs have been shown to affect drug release from the NLCs. Furthermore, the composition of the dosage form including polymer degradation and diffusion of the drug from the matrix governs drug release [87, 88]. HEC which is a controlled-release polymer might have also contributed to the slow and sustained release of the drug [89].

#### Drug release mechanism

The mechanism of drug release from the buccal films was determined by fitting the release data into several release kinetics models including zero-order, first-order, Weibull, Hixson-Crowell, Korsmeyer-Peppas, and Higuchi models. The release kinetics parameters and regression coefficients were calculated, and the Weibull model was the best fit for the data, with an adjusted R2 value of 0.9984 (Fig. 14). This model is an empirical and generalized form of the exponential function and often used to describe drug release from nanoparticles [90, 91]. The Weibull model is expressed as follows.

$$F= 100{\left.\left(1- e-\frac{(t-Ti}{\boldsymbol{\alpha }}\right.\right)}^{\beta }$$

where *F represents the fraction (%) of drug released in time t, Ti represents the lag time before the start of the dissolution or release process, which is usually near zero, β is the shape parameter that characterizes the curve, and α is the scale parameter that defines the time scale of the process *[55]*.*

**Fig. 15:**
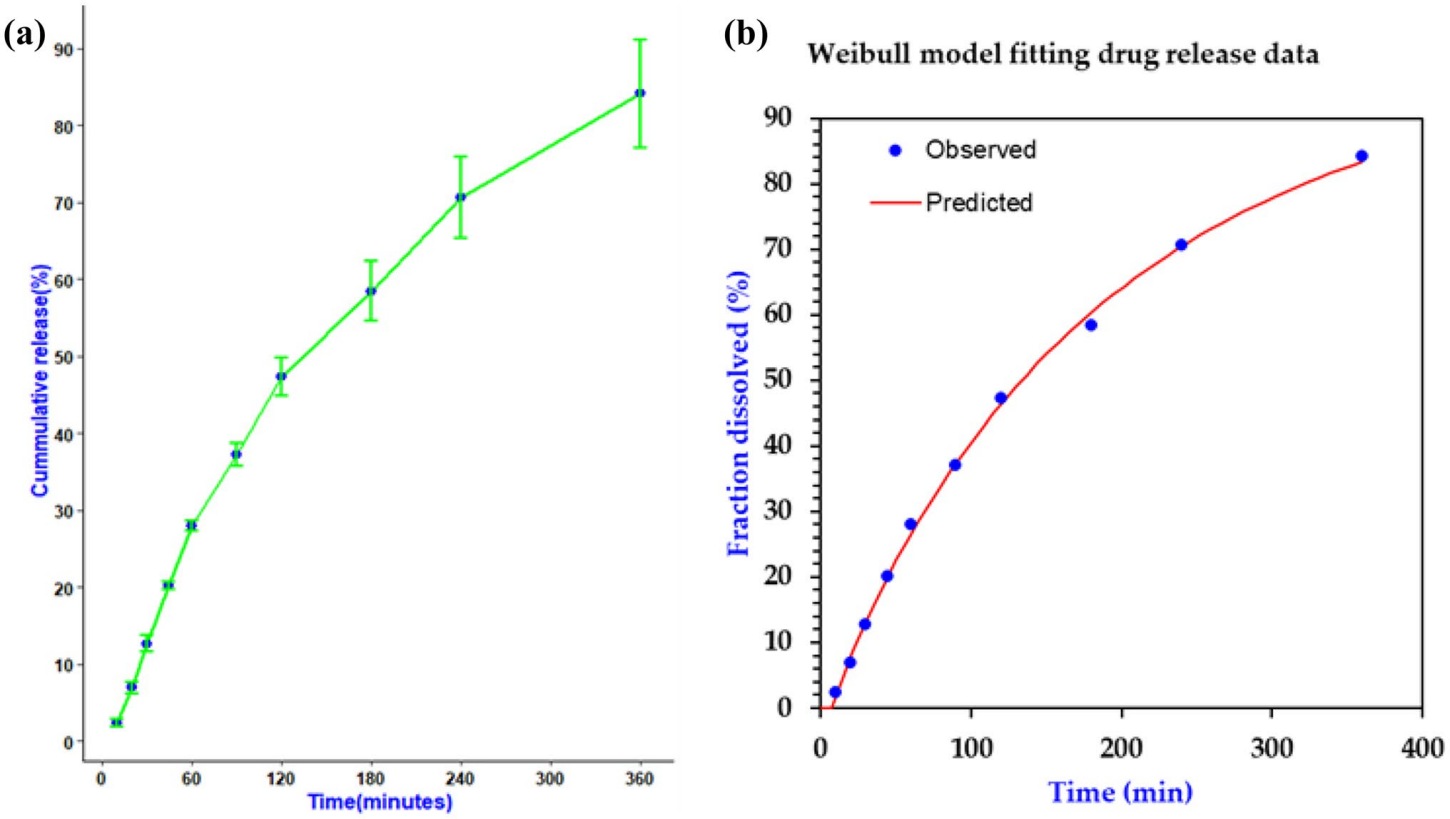
A release data of 3D printed CBD film **a** and release data fitted to Weibull model (**b**)

The drug release from the printed film has a β of 0.925, indicating a combined mechanism of Case II transport and Fickian diffusion. Values of β less than 0.75 indicate Fickian diffusion, while values between 0.75 and 1 illustrate a combined mechanism, and values higher than 1 indicate a complex release mechanism [92]. From the result, the release of CBD from the 3D printed film follows a combination of Case II transport and Fickian diffusion (Fig. 15).

### Prediction of in vivo performance

**Fig. 16:**
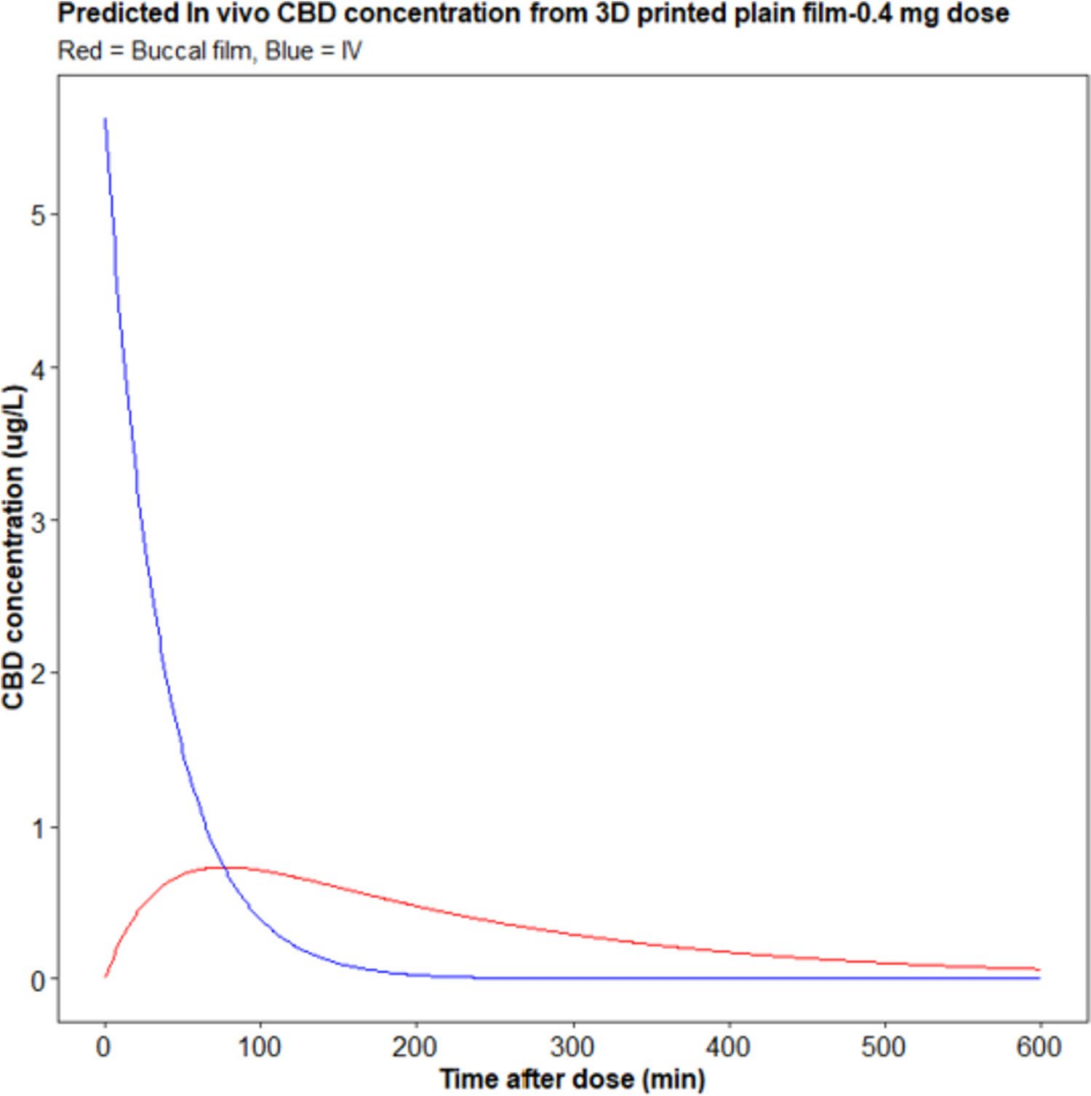
Plasma-time profile (predicted) of Cannabidiol film

In vitro-in vivo correlations (IVIVCs) are mathematical models that predict the relationship between plasma concentration and in vitro dissolution for a specific drug. These models can serve as a substitute for in vivo bioavailability studies, which can be expensive and time-consuming [93]. By developing IVIVCs, it is possible to reduce the number of animal and human bioavailability studies required during the formulation design and optimization process, as recommended by regulatory agencies such as the FDA [94]. The convolution method is a commonly used approach for IVIVC and predicts blood drug levels using in vitro dissolution data. In this study, the plasma concentration–time profile of IV CBD was used to calculate the unit input response (UIR) [95]. The predicted AUC_0–10 h_, C_max_, and T_max_ for cannabidiol film (0.4 mg) assuming 100% bioavailability were 201.5 µg·h/L, 0.74 µg/L, and 1.28 h, respectively (Fig. 16). Previous studies that reported the pharmacokinetics of Sativex oromucosal spray showed that AUC and C_max_ are dose dependent. The predicted AUC and C_max_ for the film were higher than equivalent dose of Sativex which could be due to difference in drug delivery system and higher bioavailability (100%) assumption made in our model. The predicted T_max_ (1.28 h) is comparable with previous reports of CBD T_max_ in Sativex which was 3.7 h [96].

## Conclusions and future directions

This study demonstrates the potential of 3D printing technology in producing a novel dosage form of CBD for personalized therapy. The 3D printed buccal films containing CBD-NLCs showed promising physicochemical properties, such as good flexibility, strength, and sustained drug release. The release of the drug from the film was slow and continuous release (84. 11 ± 7.02% in 6 h). The predicted in vivo concentration was 201.5 µg·h/L, 074 µg/L, and 1.28 h for AUC_0–10 h_, C_max_, and T_max_, respectively.

This innovative approach could potentially revolutionize medicine production and personalized therapy, enabling the creation of custom dosage forms with different geometries and release kinetics. Moreover, the 3D-printed buccal films with CBD-NLCs offer a promising solution to overcome the challenges associated with the poor solubility, low bioavailability, and variable pharmacokinetics of CBD.

Further studies are needed to evaluate the bioavailability and efficacy of the 3D-printed buccal films containing CBD-NLCs in appropriate models. Nonetheless, the findings of this study pave the way for the development of personalized and effective treatments for various diseases using 3D printing technology.

### Supplementary Information

Below is the link to the electronic supplementary material.

Supplementary file1 (DOC 62 KB)

## Data Availability

The datasets generated during and/or analysed during the current study are available from the corresponding author on reasonable request.
