# Supplementary material for: Combining the potential of 3D printed buccal films and nanostructured lipid carriers for personalised cannabidiol delivery
Source: Drug Deliv Transl Res. 2023 Oct 30;14(4):984–1004. doi: 10.1007/s13346-023-01446-0 (PMC10927780; doi:10.1007/s13346-023-01446-0)
Supplement: Supplementary file 1 — Supplementary file1 (DOC 62 KB) [file 13346_2023_1446_MOESM1_ESM.doc]

**Table S1.** Release parameters of fitted experimental data for CBD-NLCs 3D Printed Film


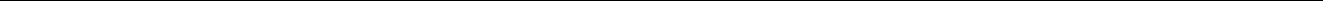


| **Model name** | **Equation** | **Goodness of** | **CBD-NLCs film** |
| --- | --- | --- | --- |
|  |  | **fit parameter** |  |
|  |  |  |  |
|  |  | *R*^2^ adjusted | 0.8734 |
| Zero order | ***F* = *k*0 × *t*** | RMSE | 9.91 |
|  |  | AIC | 69.84 |
|  |  |  |  |
|  |  | *R*^2^ adjusted | 0.9964 |
| First order | ***F* = 100 × [1 - Exp(-*k*1 × *t*)]** | RMSE | 1.66 |
|  |  | AIC | 34.13 |
|  |  |  |  |
|  |  | *R*^2^ adjusted | 0.9260 |
| Higuchi | ***F* = *k*H × *t*^0.5^** | RMSE | 7.58 |
|  |  | AIC | 64.48 |
|  |  |  |  |
| Korsmeyer- |  | *R*^2^ adjusted | 0.9752 |
|  | ***F* = *kk*P × *t^n^*** | RMSE | 4.39 |
| Peppas^b^ |  |  |  |
|  |  | AIC | 54.37 |
|  |  |  |  |
|  |  |  |  |
|  |  | *R*^2^ adjusted | 0.9891 |
| Hixson-Crowell | ***F* = 100 × [1 - (1 - *k*HC × *t*)^3^]** | RMSE | 2.91 |
|  |  | AIC | 45.33 |
|  |  |  |  |
|  |  | *R*^2^ adjusted | 0.9960 |
| Hopfenberg | ***F* = 100 × [1 - (1 - *k*HB × *t*)*^n^*]** | RMSE | 1.76 |
|  |  | AIC | 36.09 |
|  |  |  |  |
|  |  | *R*^2^ adjusted | 0.8760 |
| Baker-Lonsdale | **3/2 × [1 - (1 - *F*/100)^2/3^] - *F*/100 = *k*BL× *t*** | RMSE | 9.81 |
|  |  | AIC | 69.64 |
|  |  |  |  |
|  |  | *R*^2^ adjusted | 0.9960 |
| Peppas-Sahlin^c^ | ***F* = *k*1 × *t^m^* + *k*2 × *t*^2^*^m^*** | RMSE | 1.76 |
|  |  | AIC | 36.83 |
|  |  |  |  |
|  |  | *R*^2^ adjusted | 0.9984 |
| Weibull | ***F* = 100 × {1 - Exp[-((*t* - *Ti*)*^β^*)/*α*]}** | RMSE | 1.1031 |
|  |  | AIC | 27.42 |
|  |  |  |  |


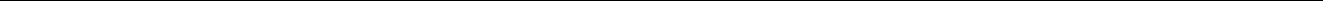


**Notes:** *F*, Percentage of drug released at time *t*; *k*0, Zero order release constant; *k*1, First order release constant; *k*H, Higuchi release constant; *k*kP, Release rate constant, and ^b^*n*, diffusional release exponent; *k*HC, Release constant relevant to Hixson-Crowell model; *k*HB, Combined constant corresponding to Hopfenberg model in which *k*HB = *k*0/(*C*0 × *α*0) where *k*0, erosion rate constant, *C*0, initial drug concentration in the matrix, *α*0, initial radius for a slab/cylinder/sphere structure, and *n*, 1, 2, and 3 for the slab, cylinder, and sphere structure, respectively; *k*BL, Combined constant related to Baker-Lonsdale model in which *k*BL = [3 × *D* × *Cs*/(*r*0^2^ × *C*0)] where *D*, diffusion coefficient, *Cs*, saturation solubility, *r*0, initial radius for a sphere/cylinder/slab structure, and *C*0, initial drug concentration in the matrix; ^c^*k*1, Constant relevant to the Fickian kinetics, and ^c^*k*2, constant relevant to Case-II relaxation kinetics, and ^c^*m*, diffusional release exponent;

**Abbreviations:** *R*^2^ adjusted, adjusted coefficient of determination; RMSE, Root mean squared error; AIC, Akaike Information Criterion.

**14**
